# GM1 Gangliosidosis—A Mini-Review

**DOI:** 10.3389/fgene.2021.734878

**Published:** 2021-09-03

**Authors:** Elena-Raluca Nicoli, Ida Annunziata, Alessandra d’Azzo, Frances M. Platt, Cynthia J. Tifft, Karolina M. Stepien

**Affiliations:** ^1^Glycosphingolipid and Glycoprotein Disorders Unit, Medical Genetics Branch, National Human Genome Research Institute, National Institutes of Health, Bethesda, MD, United States; ^2^Department of Genetics, St. Jude Children’s Research Hospital, Memphis, TN, United States; ^3^Department of Anatomy and Neurobiology, College of Graduate Health Sciences, University of Tennessee Health Science Center, Memphis, TN, United States; ^4^Department of Pharmacology, University of Oxford, Oxford, United Kingdom; ^5^Office of the Director, National Human Genome Research Institute, National Institutes of Health, Bethesda, MD, United States; ^6^Adult Inherited Metabolic Disorders, Salford Royal NHS Foundation Trust, Salford, United Kingdom; ^7^Division of Diabetes, Endocrinology and Gastroenterology, University of Manchester, Manchester, United Kingdom

**Keywords:** GM1 gangliosidosis, glycoconjugates metabolism, beta galactosidase, gene therapy, mouse model

## Abstract

GM1 gangliosidosis is a progressive, neurosomatic, lysosomal storage disorder caused by mutations in the *GLB1* gene encoding the enzyme β-galactosidase. Absent or reduced β-galactosidase activity leads to the accumulation of β-linked galactose-containing glycoconjugates including the glycosphingolipid (GSL) GM1-ganglioside in neuronal tissue. GM1-gangliosidosis is classified into three forms [Type I (infantile), Type II (late-infantile and juvenile), and Type III (adult)], based on the age of onset of clinical symptoms, although the disorder is really a continuum that correlates only partially with the levels of residual enzyme activity. Severe neurocognitive decline is a feature of Type I and II disease and is associated with premature mortality. Most of the disease-causing β-galactosidase mutations reported in the literature are clustered in exons 2, 6, 15, and 16 of the *GLB1* gene. So far 261 pathogenic variants have been described, missense/nonsense mutations being the most prevalent. There are five mouse models of GM1-gangliosidosis reported in the literature generated using different targeting strategies of the *Glb1* murine locus. Individual models differ in terms of age of onset of the clinical, biochemical, and pathological signs and symptoms, and overall lifespan. However, they do share the major abnormalities and neurological symptoms that are characteristic of the most severe forms of GM1-gangliosidosis. These mouse models have been used to study pathogenic mechanisms, to identify biomarkers, and to evaluate therapeutic strategies. Three *GLB1* gene therapy trials are currently recruiting Type I and Type II patients (NCT04273269, NCT03952637, and NCT04713475) and Type II and Type III patients are being recruited for a trial utilizing the glucosylceramide synthase inhibitor, venglustat (NCT04221451).

## Introduction

Glycoconjugates play a key role in cellular function and are tightly regulated both in terms of biosynthesis and catabolism. β-Galactosidase (β*-*GAL) is a lysosomal hydrolase that cleaves β-linked galactose residues from the non-reducing end of glycan moieties found in various glycoconjugates. Reduction in β-GAL activity leads to the accumulation of GM1 ganglioside and its asialo derivative GA1, primarily in lysosomes of neuronal tissue (Caciotti et al., 2011; Jarnes Utz et al., 2017). The first description of GM1 and GA1 storage was made in a case of amaurotic idiocy (as it was then called), now called GM1 gangliosidosis (Jatzkewitz and Sandhoff, 1963). In addition to the storage of GM1 ganglioside, other glycoconjugates with β-galactose at the non-reducing end are detectable in high concentration in patients’ urine, including N-linked glycans, and various O-linked glycans (Lawrence et al., 2019). These disease-related β-linked galactose-terminal oligosaccharides arise from the lysosomal breakdown of glycoproteins that are stored in the brain (Tsay and Dawson, 1976), liver (Holmes and O’Brien, 1978), and other biological fluids including urine (Brunetti-Pierri and Scaglia, 2008; Bruggink et al., 2012) and amniotic fluid (Piraud et al., 2017). The significant proportion of these soluble glycans are metabolites of incompletely degraded *N*-linked glycans, such as A1G1, A2G2, A3G3, and A4G4. The *N*-glycan metabolite A2G2 was proposed to be a surrogate glycan biomarker of GM1 gangliosidosis (Lawrence et al., 2019).

GM1 gangliosidosis is an example of a family of inherited metabolic disorders termed lysosomal storage diseases. The estimated incidence of GM1 gangliosidosis is 1:100,000–200,000 live births (Brunetti-Pierri and Scaglia, 2008). However, some forms of the disease appear to be more prevalent in specific geographical areas, including Southern Brazil and Japan and among the Roma (Yoshida et al., 1991; Severini et al., 1999; Sinigerska et al., 2006).

**FIGURE 1:**
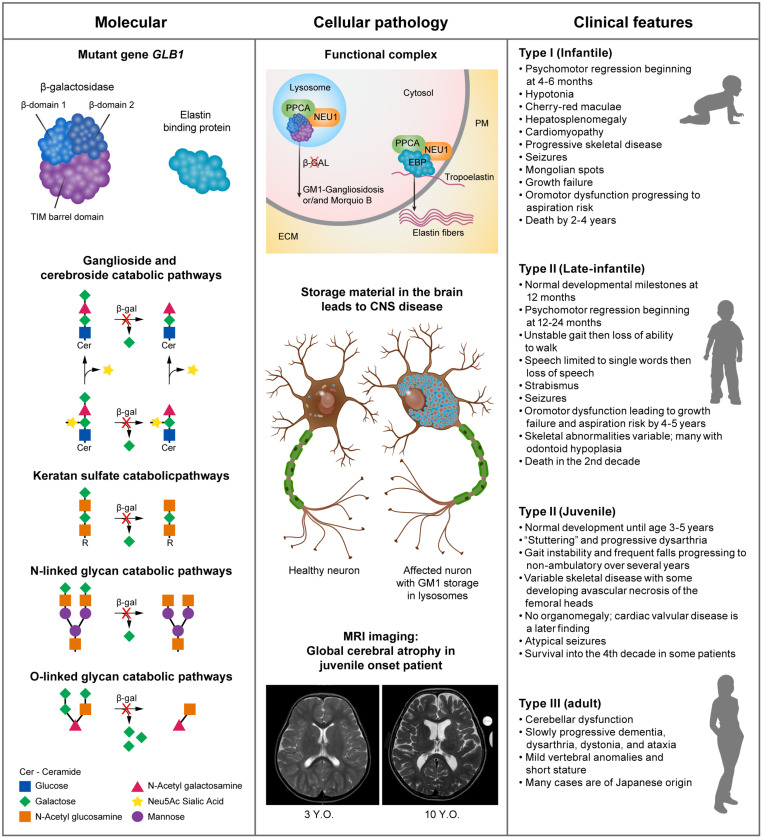
Pathogenesis and clinical manifestations. Panel 1: human β-GAL is composed of a catalytic TIM barrel domain followed by β-domain 1 and β-domain 2 (Ohto et al., 2012). Mutations in the *GLB1* gene lead to impaired enzyme activity, which results in the progressive accumulation of complex gangliosides, specifically GM1. This, in turn, initiates a series of pathogenic events that ultimately lead to neurodegeneration (Kolter, 2012; Annunziata et al., 2018). Panel 2: through alternative splicing, the *GLB1* gene gives rise to two transcripts, one encoding the hydrolytic enzyme β-galactosidase and the other the elastin binding protein (EBP). The primary role of EBP is to chaperone the deposition of elastin fibers in the extracellular matrix (ECM). β-galactosidase (GLB1) and EBP are found in complex with PPCA and NEU1 in lysosomes and the plasma membrane (PM), respectively (Caciotti et al., 2005; Bonten et al., 2014). Panel 3: although GM1 gangliosidosis is a disease continuum it can be loosely divided into 3 types, with Type II having 2 subtypes. The common use of a synthetic fluorogenic substrate to measure β-GAL activity makes it difficult to establish an accurate correlation between residual enzyme activity and clinical outcome. This may also be complicated by the regulatory and post posttranslational mechanisms that influence GM1-ganglioside catabolism and may vary among patients (Breiden and Sandhoff, 2019). The main symptoms of the disease commonly found in each type/subtype are summarized.

GM1 presents with a continuum of disease severity but patients are loosely classified based on the age of onset of the symptoms into Type I (infantile), Type II (late-infantile and juvenile) and Type III (adult). In principle, disease severity should inversely correlate with residual enzyme activity levels. However, β-GAL activity is mostly measured with the synthetic fluorogenic substrate 4-methyl-umbelliferyl-β-D-galactopyranoside, which may not be accurate enough to estimate the clinical course of the disease on the basis of the measured residual enzyme activity (Inui et al., 1990; Caciotti et al., 2011; Ferreira and Gahl, 2017; Figure 1).

**FIGURE 2:**
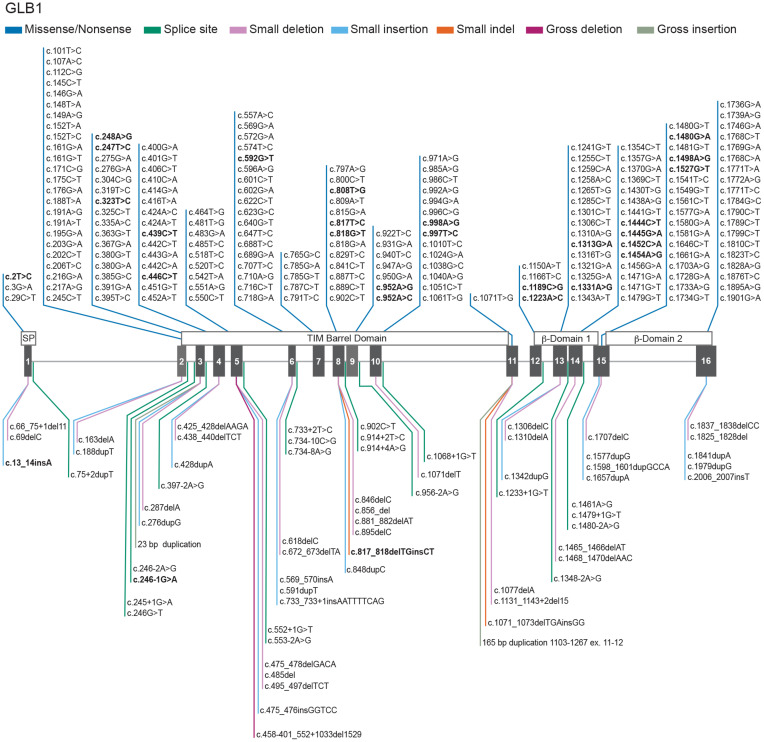
Genotypes in GM1 gangliosidosis. Schematic representation of 261 *GLB1* variants with a reported phenotype of GM1-gangliosidosis and/or Morquio B registered in the database HGMD (2021) updated with the novel variants from Tebani et al. (2021). The *GLB1* gene is located on the short arm of chromosome 3 (3p21.33). GM1 gangliosidosis and Morquio B disease result from biallelic mutations in *GLB1* gene. Mutations affecting the catalytical site of the β-GAL enzyme may reduce or eliminate the degradation of GM1 substrate. *GLB1* cDNA sequence NM_000404.4; Ref SeqGene NG_009005.1; 194 Missense/Nonsense (dark blue), 20 Splicing substitutions (dark green), 25 small deletions (pink), 17 small insertion/duplications (light blue), 2 small indels (orange), 1 gross deletion (purple), 2 gross insertion/duplications (light green). Bold text represents GM1 gangliosidosis and/or Morquio B reported phenotypes. A summary of the 261 reported variants with a phenotype of GM1 gangliosidosis and/or Morquio B disease can be found at ***GLB1*** (HGMD, 2021; Tebani et al., 2021). The main domains of the protein are shown above the exons. For a full description on the clinical pathogenicity of each variant the database ClinVar (Landrum et al., 2018) provides information about genomic variation and its relationship to human health at ***GLB1*** (ClinVar, 2021).

Bi-allelic mutations in *GLB1* result in a reduction in β-GAL activity and the build-up of GM1 ganglioside in multiple tissues including the brain (Brunetti-Pierri and Scaglia, 2008; Karimzadeh et al., 2017; Rha et al., 2021) leading to severe neurodegeneration resulting in morbidity and premature mortality (Ferreira and Gahl, 2017). More than 200 disease-causing mutations have been identified across the *GLB1* gene, particularly in exons 2, 6, 15, and 16 (Figure 2).

A smaller subset of mutations predominantly near the 3′ end of *GLB1* can result in another lysosomal storage disease, Morquio B disease (mucopolysaccharidosis type IVB), which is characterized by primary lysosomal accumulation of the glycosaminoglycan keratan sulfate. Morquio B patients have predominantly skeletal abnormalities without the neurodegenerative aspects of GM1 gangliosidosis (Ferreira and Gahl, 2017). Cases with phenotypic features of both GM1 gangliosidosis and Morquio B disease have also been described (Mayer et al., 2009).

Distinct *GLB1* gene mutations that affect the affinity of the enzyme for the two main substrates, GM1 ganglioside and keratan sulfate, explain at least in part the different clinical manifestations and the degree and type of storage material (Figure 2).

Five murine models have been developed that mimic the human phenotype and have formed that basis of our current understanding of disease pathogenesis. They have also been instrumental in the trialing of new therapies.

Comprehensive reviews of GM1 gangliosidosis have been recently published (Regier et al., 1993; Breiden and Sandhoff, 2019; Lawrence et al., 2019; Lang et al., 2020; Rha et al., 2021). In this mini review, we will focus on the clinical spectrum of disease and genetic variants, our current understanding of disease pathogenesis, the utility of available animal models and approaches to therapy for these devastating disorders.

## Clinical Manifestations

Among the three subtypes of GM1 gangliosidosis, the infantile form is the most severe, with onset of symptoms before 6 months of age and death early in childhood (Jarnes Utz et al., 2017; Lang et al., 2020). One of the earliest signs that can be seen in infantile disease prenatally is hydrops fetalis, which is associated with many severe cases of LSDs (Stone and Sidransky, 1999), and should stimulate an investigation into the underlying causes of this phenotype (Iyer et al., 2021). The early diagnosis of infantile onset disease would have the greatest chance of successful therapeutic intervention.

Type II consists of late infantile and juvenile subtypes (Figure 1). Late infantile disease patients meet developmental milestones at 12 months but have onset of symptoms generally between 12 and 24 months. Patients quickly lose the ability to ambulate and have difficulty swallowing and handling secretions necessitating gastrostomy placement. Late infantile patients succumb to their illness by the mid second decade.

Juvenile patients have onset at 3–5 years of age, having learned to walk, run, and speak in sentences. The first symptoms may be unsteady gait, frequent falls, and “slurring” of speech progressing to ataxia and dysarthria. Children become unable to walk without assistance then lose the ability to walk. Dysarthria progresses to inability to swallow effectively and subsequent weight loss, necessitating gastrostomy tube placement. Children with juvenile onset disease can live into their 4th decade of life (Regier et al., 1993, 2016; Jarnes Utz et al., 2017).

Adult onset GM1 represents with an attenuated form with slower disease progression and very mild dysmorphic features (Suzuki et al., 2001) and greater clinical variability (Tonin et al., 2019). The onset of symptoms occurs in early childhood to late teens, as described in individuals of Japanese descent (Inui et al., 1990; Arash-Kaps et al., 2019).

Patients with all types of GM1 gangliosidosis can have variable skeletal disease including pectus carinatum with spine and rib changes that lead to restrictive lung disease (Ferreira et al., 2020). Radiographic changes can allow differentiation between the late-infantile and juvenile forms of Type II disease. Odontoid hypoplasia, as observed in all late infantile patients but not juvenile patients, requires cervical spine evaluation in late infantile patients prior to anesthesia, and anesthetic care optimally provided by pediatric anesthesiologists with experience in patients with cervical spine pathology to prevent perioperative morbidity or mortality (Ferreira et al., 2020).

The progressive nature of the neurological disease in GM1 gangliosidosis requires close monitoring. Biomarkers in blood, urine, and CSF biomarkers may be potentially useful in this regard (Gray-Edwards et al., 2017a,b; Rha et al., 2021). Disease progression can also be monitored using minimally invasive neuroimaging. In Type II disease both late infantile and juvenile patients demonstrate progressive atrophy in the cerebrum and cerebellum with greater variability and slower progression in the juvenile subtype. Quantitative magnetic resonance spectroscopy (MRS) shows increasing deficits of *N*-acetyl aspartate (NAA) across many brain regions with greater and swifter deficits seen in the more rapidly progressive late infantile subtype (Regier et al., 2016). These biomarkers of disease progression also correlate with developmental progression of disease and may serve as useful outcome measures for clinical trials.

In contrast to GM1 gangliosidosis, Morquio B disease (Figure 2) patients do not have CNS disease but can have neurologic compromise due to underlying skeletal disease, such as spinal nerve compression (Abumansour et al., 2020).

## Genotypes

There is a poor genotype-phenotype correlation in GM1 gangliosidosis demonstrated by the clinical variability in age of onset and progression of disease even between siblings with the same genotype. One can speculate that polymorphisms or mutations in the other genes of the β-GAL complex, protective protein/cathepsin A (PPCA) and neuraminidase 1 (NEU1) (see Figure 1) may account for this variability. An additional limitation to reach an accurate genotype-phenotype correlation is posed by the way the enzyme assay is commonly performed using a soluble fluorogenic substrate that does not reflect the topology and membrane microenvironment of the natural substrate, GM1 ganglioside. Moreover, regulatory, and post-translational mechanisms that modulate GM1 catabolism further hamper an accurate prediction of the clinical course of the disease in patients, if based only on residual enzyme activity against the synthetic substrate (Breiden and Sandhoff, 2019).

Most patients with GM1 gangliosidosis are compound heterozygotes and aside from biallelic null mutations that produce type I disease, it is difficult to attribute specific phenotypes to any single mutation. Generalizations based on crystallographic structure of the β-GAL enzyme have been attempted (Ohto et al., 2012). Mutations associated with type I/infantile onset GM1 gangliosidosis, for the most part, are located in the core protein region causing β-gal instability, whereas mutations associated with milder phenotypes, such as types II and III GM1 gangliosidosis, tend to be on the protein surface (Morita et al., 2009; Ohto et al., 2012; Rha et al., 2021). Recently, the determination of the 3D structure of murine β-gal in complex with PPCA has revealed that some mutations at conserved amino acid residues found in GM1 gangliosidosis patients affect formation of the complex (Gorelik et al., 2021). These findings further complicate genotype-phenotype correlation, in relation to the penetrance of specific disease phenotypes.

Out of the total 261 reported pathogenic variants associated with a phenotype of GM1 gangliosidosis and/or Morquio B disease, most of them are missense/nonsense (194), and the rest are splicing substitutions (20), small deletions (25), small insertion/duplications (17), small indels (2), gross insertion/duplications (2), and a single large deletion (Figure 2). The largest number of mutations are found in exons 2 (26 variants), 6 (23 variants), 15 (21 variants) and 16 (24 variants). Previous reports implicate exons 2, 6, and 15 (Brunetti-Pierri and Scaglia, 2008) as hot spots for mutations, however exon 16 also harbors multiple pathogenic variants.

## Mouse Models

The first two genetically engineered mouse models of GM1 gangliosidosis were reported in 1997 by two groups (Hahn et al., 1997) and (Matsuda et al., 1997a,b). These knockout mice were generated by homologous recombination at the murine *Glb1* locus that disrupted the gene by introducing a selectable marker cassette in either exon 6 or exon 15 (Hahn et al., 1997; Matsuda et al., 1997a,b). Both mouse models closely recapitulated the infantile/juvenile onset form of GM1 gangliosidosis and have been used extensively for studying disease pathogenesis and for testing therapeutic modalities (Table 1).

**TABLE 1:** Overview of developed GM1 gangliosidosis mouse models: characteristics and therapies tested.

	Genotype	*Glb1^–/–^* or *Glb1*^*tm1Adz*^/knock-out (Hahn et al., 1997)	*Glb1^–/–^* or *Glb1*^*tm1Jmat*^/knock-out (Matsuda et al., 1997a,b)	*Glb1^–/–^* or β -*gal^–/–^*/knock-out (Przybilla et al., 2019)	*Glb1^–/–^*/knock-out (Eikelberg et al., 2020)	*Glb1*^(G455R)^/knock-i (Liu et al., 2021)
	Exon targeted	6	15	8	15	14
	Technology used to generate	Homologous recombination and embryonic stem cell technology	Homologous recombination and embryonic stem cell technology	CRISPR/Cas9	TALEN	CRISPR/Cas9
Evaluations	Life span	∼6–7 months	∼7–10 months	∼10 months	∼8 months is the last experimental timepoint reported	∼11 months
	Gross neurological and behavioral symptoms	∼4–5 months	∼6–8 months	∼6 months	∼3.5–4 months	∼4–8 months
	β-gal activity	∼0–2%	∼0–10%	∼0–13%	∼0–12%	∼0–3%
	mRNA status	Absent	n/a	n/a	Shortened mRNA	Retention of mutant mRNA
	GM1 levels compared to control	∼2–7×	∼4–6	∼2–4%	∼2–15×	∼4×
	Histopathology and morphologic analyses	At 3 weeks of age, swollen neurons containing storage material. At the EM level, neurons at the same age show pleiomorphic inclusions	Vacuolated lymphocytes in peripheral blood. No evident skeletal dysplasia. Degenerated neurons with distended cytoplasm and multilamellar and myelin-like inclusion bodies.	Impaired neurocognitive function (Barnes maze and spontaneous alteration T-maze tests)	Axonopathy and reduction of membrane resistance (electrophysiology and single cell electroporation experiments).	Inflammatory response and abnormal autophagy in the brains: CNS inflammation with activated microglia and abnormal autophagy
	Therapy	• ERT (Chen et al., 2020) • SRT (Jeyakumar et al., 2003; Kasperzyk et al., 2004, 2005; Elliot-Smith et al., 2008) • *Ex vivo* gene therapy (Sano et al., 2005) • *In vivo* gene therapy (Broekman et al., 2007; Baek et al., 2010; Weismann et al., 2015; Hinderer et al., 2020)	• ERT (Matsuda et al., 2003; Suzuki, 2006, 2008; Suzuki et al., 2007, 2012; Takai et al., 2013) • *In vivo* gene therapy (Takaura et al., 2003)	• ERT (Przybilla et al., 2021)	n/a	n/a

The β-*gal^–/–^* mice described by Hahn et al. (1997) showed substantial early neuronal loss in the brain and spinal cord (Tessitore et al., 2004). GM1 is abundant in the neuronal plasma membrane (PM) and is the only ganglioside that can influence Ca^2++^ transfer across membranes by interacting with Ca^2+^-binding proteins (Ledeen and Wu, 2015; Annunziata et al., 2018). In β-*gal^–/–^* neurons impaired lysosomal degradation of GM1 results in the abnormal accumulation of the ganglioside in internal membranes, specifically those of the ER resulting in two pathogenic effects: (1) it enhances the flux of Ca^2+^ out of the ER, thereby altering ER Ca^2+^ levels, which activates an unfolded protein response (UPR) (Tessitore et al., 2004); and (2) it increases the number of membrane contact sites between the ER and mitochondria, known as mitochondria associated ER membranes (MAMs). These GM1-enriched microdomains mediate the abnormal flux of Ca^2+^ from the ER to the mitochondria, which ultimately results in mitochondria Ca^2+^ overload (Sano et al., 2009). The combination of these Ca^2+^-dependent pathogenic events steers the simultaneous activation of UPR and mitochondria-mediated neuronal apoptosis (Tessitore et al., 2004; Sano et al., 2009). In addition, the progressive neurodegeneration in these mice elicits a widespread neuroinflammatory response, accompanied by the release of cytokines and chemokines in the brain interstitial fluid and the CSF (Jeyakumar et al., 2003; Sano et al., 2005), which likely accelerates CNS disease. This neuroinflammatory response was shown to favor the recruitment of genetically modified BM monocytes expressing a therapeutic β-*gal* enzyme, following *ex vivo* gene therapy in β-*gal^–/–^* mice (Sano et al., 2005) (see below).

With the development of new gene editing approaches, additional β-*gal* deficient mouse models have been more recently generated using either TALEN or CRISPR/Cas9 technologies (Przybilla et al., 2019; Eikelberg et al., 2020; Liu et al., 2021). Using CRISPR/Cas9 Przybilla et al. (2019) engineered a knock-out mouse model by introducing a deletion in exon 8 of the *Glb1* gene. Phenotypic alterations in these mice were evaluated using behavioral tests that showed profound neurocognitive impairment (Przybilla et al., 2019). In Eikelberg et al. (2020) described a knock-out model using TALENs to target exon 15 of *Glb1*. These mice display brain and spinal cord pathology, characterized by swelling of axons and loss of myelin, leading to abnormal electrophysiological activity of neurons (Eikelberg et al., 2020). This is the first electrophysiological study performed in a mouse model of the disease, showing abnormalities that the authors attribute to increased neuronal cell size and reduced membrane resistance. The most recent β-*gal* mutant mouse generated using CRISPR/Cas9 is a knock-in model that introduces a human missense mutation in exon 14 of *Glb1*, described in a patient with late-infantile GM1 gangliosidosis (Liu et al., 2021). The CNS phenotype in this model included impaired motor function, as well as extensive microgliosis, accompanied by activation of autophagy (Liu et al., 2021). The characteristics of available mouse models are summarized in Table 1.

## Therapeutic Strategies

Until very recently, therapy for GM1 gangliosidosis was limited to symptomatic management. However, several experimental therapies have been trialed in murine (Table 1) and feline models (Gray-Edwards et al., 2017a,b, 2020). Since GM1 primarily affects the brain, targeted delivery must traverse the blood-brain barrier (BBB) or be delivered directly to the brain. Experimental therapies are discussed below.

### Substrate Reduction Therapy

The rationale of Substrate Reduction Therapy (SRT) is to use small molecule inhibitors of enzymes responsible for the biosynthesis of stored substrates (Jeyakumar et al., 2002; Platt et al., 2018). For example miglustat, is a *N*-alkylated iminosugar that is a reversible competitive inhibitor of glucosylceramide synthase, the enzyme catalyzing the first committed step in the biosynthesis of most glycosphingolipids, including gangliosides (Platt et al., 1994). This approach aims to balance the rate of glycosphingolipid biosynthesis with the impaired rate of glycosphingolipid catabolism (Platt et al., 2001). Miglustat crosses the BBB and so can in principle be applied to treat glycosphingolipid storage diseases affecting the periphery and the brain (Cox et al., 2000; Kasperzyk et al., 2004, 2005; Patterson et al., 2007; Treiber et al., 2007; Ficicioglu, 2008). Miglustat was approved for treating type 1 Gaucher disease in 2002 and for Niemann-Pick disease type C in 2009 (Platt et al., 2018). Miglustat has also been proposed for the treatment of GM1 gangliosidosis. Indeed, miglustat reduced GM1 ganglioside in the central nervous system of a mouse model of GM1 gangliosidosis (Kasperzyk et al., 2004), and led to functional improvements and a decrease in brain inflammation (Kasperzyk et al., 2004; Treiber et al., 2007; Elliot-Smith et al., 2008).

Despite the demonstrated effectiveness of miglustat in other storage disorders, its use in GM1 gangliosidosis type II has been tested only in a few patients. In 2007, Tifft et al. (2007) reported that miglustat administration improved neurological functions in two patients with juvenile GM1 gangliosidosis (Tifft et al., 2007). Deodato et al. (2017) described similar neurological improvement in the juvenile form of the disease (Deodato et al., 2017). Stabilization and/or slowing of neurological progression in three of four patients was observed by Fischetto et al. (2020).

Miglustat combined with a ketogenic diet has been used to treat children with GM1 and GM2 gangliosidosis (ClinicalTrials.gov Identifier NCT02030015). The aim of this study was to learn if synergistic enteral regimen for treatment of the gangliosidoses will show improvement in overall survival and clinical benefits in neurodevelopmental abilities in children with gangliosidosis diseases. The Syner-G regimen may have prolonged lifespan, however, the small sample size and variability in other palliative care measures used by families prevented definitive conclusions to be drawn (Jarnes Utz et al., 2017).

Venglustat, another SRT drug chemically distinct from miglustat and designed specifically to cross the BBB is an orally available inhibitor of the enzyme glucosylceramide synthase (Peterschmitt et al., 2021). It is currently under study for GM1 gangliosidosis and several other LSDs in the same degradation pathway including late-onset GM2-gangliosidosis (Tay-Sachs and Sandhoff diseases), Fabry disease, and neuronopathic Gaucher disease (type III) (NCT04221451).

### Enzyme Enhancement Therapy

Enzyme enhancement therapy (EET), also termed pharmacological chaperone therapy, has been proposed for GM1 (Matsuda et al., 2003; Suzuki, 2006). The aim is to use small molecules to stabilize potentially unstable or misfolded mutant proteins in the endoplasmic reticulum to enhance lysosomal delivery and increase half-life (Parenti, 2009). Small molecule chaperones that cross the BBB, would be a prerequisite for disorders with CNS involvement (Begley et al., 2008).

Several pharmacological chaperones including galactose, *N*-octyl-4-epi-β-valienamine (NOEV)alkylated or fluorinated derivates of *N*-butyldeoxynojirimycin (NB-DNJ), and (5aR)-5a-C-Pentyl-4-epiisofagomine have been tested against numerous *GLB1* mutant enzymes (Matsuda et al., 2003; Suzuki et al., 2007, 2012; Suzuki, 2008, 2014; Fantur et al., 2010; Takai et al., 2013; Parenti et al., 2015; Thonhofer et al., 2016; Front et al., 2017).

Treatment with NOEV, a galactose analog, at the early stage of the disease reduced disease progression and prolonged survival in a murine model of GM1 gangliosidosis (see Table 1; Suzuki, 2014; Parenti et al., 2015). The compound was determined to cross the BBB for CNS delivery (Suzuki, 2014).

Collectively, dozens of patient cell lines with missense mutations have been shown to be responsive to the chaperones listed above, in some cases resulting in greater than the 10–15% residual β-gal activity sufficient to avoid substrate accumulation (Leinekugel et al., 1992; Iwasaki et al., 2006).

Pharmacological chaperones have broad tissue distribution and can be given orally; major advantages for treatment (Front et al., 2017). In addition, they have been shown to work synergistically with other therapies, such as ERT (Kishnani et al., 2017).

### Enzyme Replacement Therapy

Enzyme replacement therapy (ERT) as potential treatment for GM1 gangliosidosis was first tested using either a purified (Reynolds et al., 1978) or a recombinant (Samoylova et al., 2008) feline β-gal enzyme *in vitro*. Since β-gal cannot cross the BBB, several therapeutic “Trojan horse” strategies have been utilized, including creating fusion proteins of the enzyme with lectin subunit ribosome-inactivating toxin B (RTB) of ricin (Condori et al., 2016), and to the carboxyl terminus of the heavy chain of a mouse chimeric monoclonal antibody against the mouse transferrin receptor (mTfR-GLB1) (Przybilla et al., 2021).

Encapsulation of β-gal enzyme into artificial nanoparticles to traverse the BBB has also been experimented *in vitro* (Gupta et al., 2017; Kelly et al., 2017). Mechanically breaching the BBB has been described by Chen et al. (2020) who used direct intracerebroventricular (ICV) injection of rhβ-gal to β*-gal^–/–^* mice, which led to normalization of neuropathology.

### Stem Cell Transplantation

Stem cell transplantation (SCT) early in the course of disease may ameliorate symptoms in GM1 gangliosidosis (Sawada et al., 2009), although for optimal benefit, like Krabbe disease, the transplant would need to be undertaken in the first few weeks of life; a strong argument for universal newborn screening for GM1 disease. Although it could be successfully utilized in GM1 gangliosidosis and may reduce visceral features, the long-term correction of neurological symptoms is less likely. Improvement was observed in a 7-month GM1 gangliosidosis baby who after SCT developed normally until regression was noted at the age of 20–25 months (Shield et al., 2005). The risk of procedure-related mortality with transplantation has decreased with improvements in chemotherapy regimens and should be considered in cases of very early diagnosis with limited therapeutic options.

### Gene Therapy

Pre-clinical studies in mouse models resulted in extended life expectancy, β-gal activity restoration and decreased storage levels in the CNS and peripheral organs (see Table 1). After the successful treatment in the mouse (Takaura et al., 2003; Broekman et al., 2007, 2009; Baek et al., 2010; Weismann et al., 2015) studies were extended to the feline model with dramatic response in widespread distribution of β-gal enzyme, improved function, and greatly extended lifespan (McCurdy et al., 2014; Gray-Edwards et al., 2017a,b). The dramatic improvement observed in the murine and feline models paved the way for *in vitro* studies in human cerebral organoids (Latour et al., 2019) and subsequent phase I clinical trials in patients with Type I and Type II disease. Three trials are currently recruiting: intravenous delivery of AAV9-GLB1 (ClinicalTrials.gov Identifier NCT03952637), LYS-GM101 administered via cisterna magna (ClinicalTrials.gov Identifier NCT04273269), and PBGM01 delivered via cisterna magna (ClinicalTrials.gov Identifier NCT04713475).

## Conclusion

GM1 gangliosidosis is a severe LSD underpinned by complex pathophysiological mechanisms. Numerous disease causing *GLB1* mutations have been reported. However, genotype-phenotype correlation has been difficult to establish due in part to the way the enzyme is commonly assayed in patients’ samples. In addition, the clinical outcome of the disease in patients may be strongly influenced by post-translational and regulatory mechanisms controlling GM1 catabolism that may vary from patient to patient. These caveats ask for further elucidation of the cellular pathophysiology underlying this disease that may improve our understanding of the fundamental cell biology of GM1 ganglioside and the enzyme complex that regulates its catabolism in the lysosome. Multiple mouse models of this disorder have been instrumental for the pre-clinical testing of multiple therapies, several of which are currently in clinical trials.

## Author Contributions

E-RN, KS, FP, CT, Ad’A, and IA were involved in designing the concept of the review and oversight. KS and E-RN drafted the manuscript. All authors reviewed the manuscript, read, and approved the final manuscript.

## Conflict of Interest

Ad’A holds the Jewelers for Children Endowed Chair in Genetics and Gene Therapy. The remaining authors declare that the research was conducted in the absence of any commercial or financial relationships that could be construed as a potential conflict of interest.

## Publisher’s Note

All claims expressed in this article are solely those of the authors and do not necessarily represent those of their affiliated organizations, or those of the publisher, the editors and the reviewers. Any product that may be evaluated in this article, or claim that may be made by its manufacturer, is not guaranteed or endorsed by the publisher.

## Abbreviations

BBB: Blood-brain barrier
β -GAL: β-galactosidase
EBP: Elastin binding protein
PPCA: Protective protein/cathepsin A
NEU1: α -neuraminidase 1
CNS: Central nervous system
CRISPR/Cas9: Clustered regularly interspaced short palindromic repeats and CRISPR-associated protein 9
TALEN: Transcription activator-like effector nucleases
ERT: Enzyme replacement therapy
EET: Enzyme enhancement therapy
SRT: Substrate reduction therapy
AAV: Adeno-associated virus
ICV: Intracerebroventricular
mTfR: Mouse transferrin receptor
*N*B-DNJ: *N*-butyldeoxynojirimycin; miglustat
NOEV: N-octyl -4-epi-β-valienamine
HGMD: Human Gene Mutation Database
Cer: Ceramide
GlcCer: Glucosylceramide
LacCer: Lactosylceramide
GA2: Asialo-GM2 (GalNAc1,4Gal1,4Glc1Cer)
GA1: Asialo-GM1 (Gal1,3GalNAc1,4Gal1,4Glc1Cer)
GM3: Neu5Ac2,3Gal1,4Glc1Cer
GM2: GalNAc1,4(Neu5Ac2,3)Gal1,4Glc1Cer
GM1a: GM1 [Gal1,3GalNAc1,4(Neu5Ac2,3)Gal1,4Glc1Cer]
Glc: Glucose
Gal: Galactose.
